# The optimal concentration of silver nanoparticles in sterilizing fish skin grafts

**DOI:** 10.1038/s41598-022-23853-y

**Published:** 2022-11-14

**Authors:** Abdelnaby M. Elshahawy, Ghada Abd-Elmonsef Mahmoud, Doaa M. Mokhtar, Ahmed Ibrahim

**Affiliations:** 1 Department of Physics, Faculty of Science, Assiut University, Assiut, 71516 Egypt; 2 Botany and Microbiology Department, Faculty of Science, Assiut University, Assiut, 71516 Egypt; 3 Department of Cell and Tissues, Faculty of Veterinary Medicine, Assiut University, Assiut, 71526 Egypt; 4 Department of Histology and Anatomy, Badr University in Assiut, Assiut, Egypt; 5 Veterinary Teaching Hospital, Faculty of Veterinary Medicine, Assiut University, Assiut, 71526 Egypt

**Keywords:** Nanoscale biophysics, Biological techniques, Evolution, Microbiology, Zoology, Medical research, Materials science

## Abstract

Collagen integrity should be considered on using a sterilizing agent for fish skin grafts. This study defined the optimal concentration of silver nanoparticles (Ag NPs) for sterilization of fish skin grafts without disrupting collagen content based on microbiological and histological evaluation. Strips of tilapia skin (n = 5) were randomly allocated to be immersed in Ag NPs solution at different concentrations of 25, 50, 100, and 250 µg/mL, respectively, for 5 min. The treated skin strips underwent bacteriological and histological evaluation. Yeast and fungi were more sensitive to Ag NPs than bacteria. On increasing the nanoparticles concentration, the total counts of aerobic bacteria decrease giving 933.3 ± 28.67, 601 ± 27.66, 288 ± 16.8, 15 ± 4.08 (CFU/cm^2^ ± S.D) at 25, 50, 100, and 250 µg/mL, respectively, comparing with untreated sample (1453.3 ± 57.92). Yeasts and filamentous fungi also exhibited a similar response, achieving a complete inhibition at 100 and 250 µg/mL. *Bacillus cereus* and *Escherichia coli* were the dominant aerobic bacteria*, Candida albicans* and *Rhodotorula glutinis* were the dominant aerobic yeasts, whereas *Aspergillus niger, Aspergillus fumigatus,* and *Rhizopus stolonifer* were the dominant aerobic fungi. The collagen fibers were loose with a wavey pattern at 25 µg/mL, wavey and slightly disorganized at 50 µg/mL, highly disorganized at 100 µg/mL, and compactly arranged and slightly loose at 250 µg/mL. Ag NPs at a concentration of 250 µg/mL could be considered a reliable and feasible method for the sterilization of fish skin grafts before application on human skin with an effective antimicrobial effect and less disrupting impact on collagen content.

## Introduction

Tilapia skin is a well-known biological material used as an occlusive dressing for burn healing. It is also a cheap and important source of type I collagen. Additionally, it was confirmed that it contains non-infectious microbiota^1^, and has a morphological structure homologous to the human skin^2^. Tilapia skin is also a highly available product, safe, innovative, and easy to apply materials. Thus, it could be suggested as a potential xenograft for treating burns and regenerative medicine^3^.

Due to their numerous applications, silver nanoparticles (Ag NPs) are one of the largest and most commonly synthesized nanomaterials, they are unique antimicrobial agents and are used in various commercial medications^4^. Ag NPs have got a lot of attention as a specialized tool for managing a variety of bacterial, fungal, and viral illnesses, especially with the rise of antibiotic-resistant microorganisms^5^. The nanoparticles' tiny size allows for improved contact with the bacterial cell as well as easier penetration causing the cell to generate a high amount of reactive oxygen species (ROS) for defense^6,7^. ROS is a molecule that causes cell death and can kill cells in one of two ways: apoptosis or necrosis^8–10^. Nano-silver can interact with cell components such as proteins, lipids, enzymes, and DNA once it has been pierced by bacteria. This interaction causes significant damage to bacterial cells, including malfunction and eventual death, especially with bacterial cell ribosomes that produce ribosome denaturation, and inhibits protein synthesis^11^. Silver nanoparticles have antibacterial effects against common fish and animal pathogenic bacteria like *Aeromonas hydrophila, Aeromonas salmonoid, Aeromonas bestiarum, Klebsiella pneumoniae, Pseudomonas flourescens, Vibrio harveyi, Micrococcus luteus, Proteus* spp., *Staphylococcus aureus*, and *Flavobacterium* spp.^12–18^.

It is urgent to sterilize fish skin grafts before their application to human skin. However, collagen integrity should be considered in the sterilization of fish skin grafts along with the efficient antimicrobial effect of the sterilizing agent. In a previous study, Ibrahim et al.^3^, investigated that the silver nanoparticles (Ag NPs) explored advantage over both; chlorhexidine gluconate 4% and povidone iodine 10% in sterilizing of fish skin based on the microbial count and the collagen fiber integrity. However, this study had a limited use of one concentration of Ag NPs (25 µg/mL).

Therefore, this study represents a continuation of the previous work, aimed to evaluate different concentrations of the Ag NPs to determine the idealistic concentration for sterilizing fish skin grafts without disrupting their collagen content based on microbiological and histological evaluations.

## Results

### Microbiological analysis

**Figure 1:**
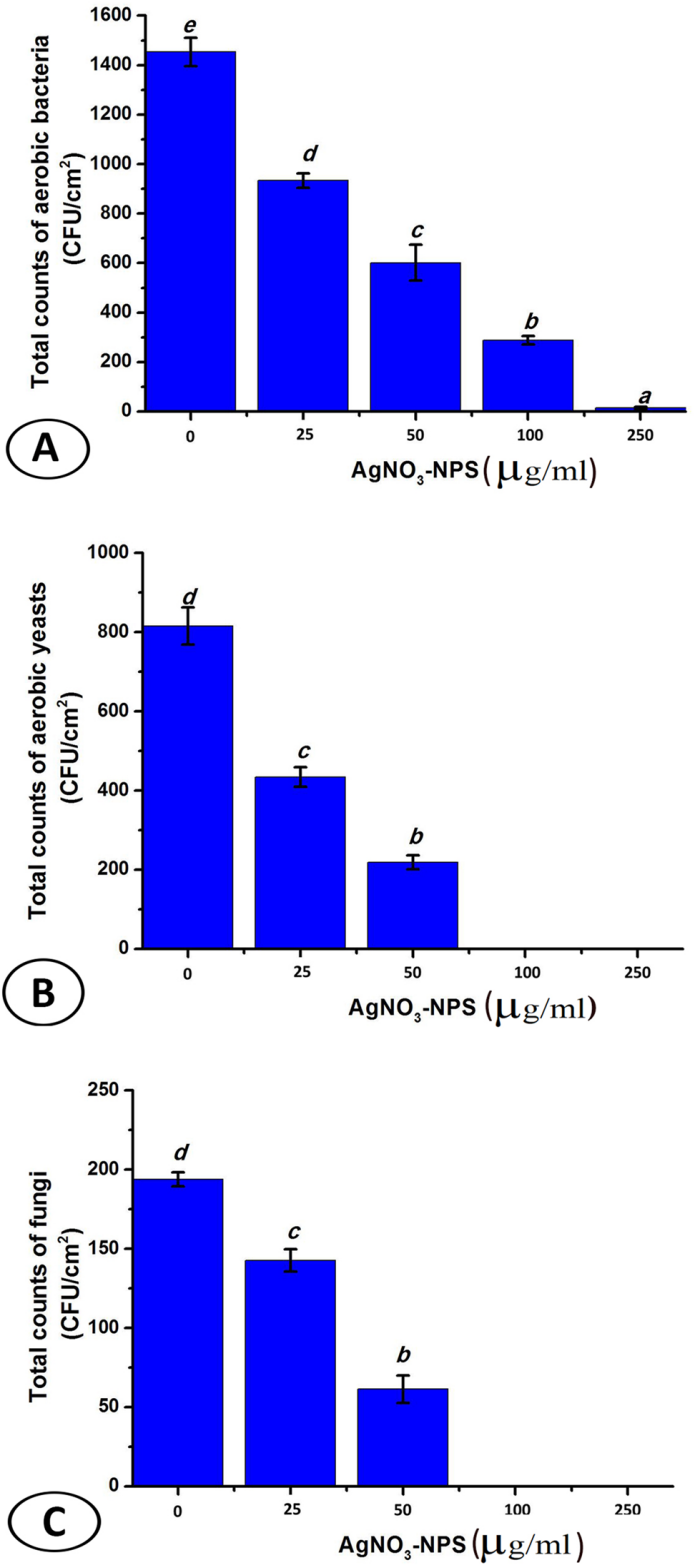
Total accounts (CFU/cm^2^ ± SD) of aerobic bacteria (**A**), aerobic yeasts (**B**), and aerobic filamentous fungi (**C**) on fish skin sterilized with different concentration of Ag NPS (0, 25, 50, 100, 250 µg/mL).

The inhibiting effect of different concentrations of Ag NPs (25, 50, 100, and 250 µg/mL) on the microbial growth of aerobic bacteria, yeasts, and filamentous fungi are shown in Fig. 1A–C. It was clear that yeast and fungi were more sensitive to Ag NPs than bacteria. For bacteria, on increasing the nanoparticles concentration, the total counts of aerobic bacteria decrease giving 933.3 ± 28.67, 601 ± 27.66, 288 ± 16.8, 15 ± 4.08 (CFU/cm^2^ ± S.D) at 25, 50, 100, and 250 µg/mL, respectively, comparing with the untreated sample (1453.3 ± 57.92) (Fig. 1A). Yeasts and filamentous fungi also exhibited a similar response, achieving a complete inhibition at 100 and 250 µg/mL, while giving 434.3 ± 24.3 and 218 ± 17.9 for aerobic yeasts and 142.7 ± 7.13 and 61.3 ± 8.6 for aerobic fungi at 25 and 50 µg/mL, respectively, comparing with the untreated sample (816 ± 47.3 and 194 ± 4.3) for yeasts and fungi, respectively (Fig. 1B, C). On examining the microbial species isolated from the fish skin; *Bacillus cereus* and *Escherichia coli were the dominant aerobic bacteria, Candida albicans* and *Rhodotorula glutinis* were the dominant aerobic yeasts, while *Aspergillus niger, Aspergillus fumigatus,* and *Rhizopus stolonifer* were the dominant aerobic fungi as shown in Table 1.

**Table 1:** Some physiological and biochemical characteristics of *Candida albicans, Rhodotorula glutinis, Bacillus cereus,* and *Escherichia coli.*

Characteristic of yeast isolates	*Candida albicans*	*Rhodotorula glutinis*	Characteristic of bacterial isolates	*Bacillus cereus*	*Escherichia coli*
Shape	Ovoid	Ovoid	Shape	bacilli	Long rod
Color	White to cream	Orange-red	Gram stain	+ve	−ve
Surface	Smooth	Smooth,glossy	Growth on MacConkey agar	−	+
Margin	Entire	Entire	Spore formation	+	−
Budding	+	Multilateral	Motility test	+	+
Formation of: Pseudohyphae	+	−	Ryu’s method	Watery	Threads
Mycelium	−	−	Hydrolysis of Starch	+	+
Arthroconidia	−	−	Gelatin	+	−
Ballistoconidia	+	−	Casein	+	+
Chlamydospore	−	−	Esculin	+	−
Endospore	−	−	Urea	−	−
Germ tube	+	−	Catalase	+	+
Glucose fermentation	+	−	Oxidase	−	−
Carbon source utilization:			Indol production	−	+
l-Arabinose	+	−	Methyl red test	−	+
d-Fructose	+	+	Voges–Proskauer tests	−	−
d-Glucose	+	+	Nitrate reduction	+	+
d-Galactose	+	+	H_2_S production	−	−
d-Xylose	+	+	Carbon sources used for growth:		
l-Sorbose	+	−	Fructose	+	−
Glycerol	+	+	Glucose	+	+
Sucrose	−	+	Lactose	+	+
Starch	+	+	Maltose	+	−
Lactose	−	−	Mannitol	+	+
Maltose	+	+	Sucrose	+	+

### Histological analysis

**Figure 2:**
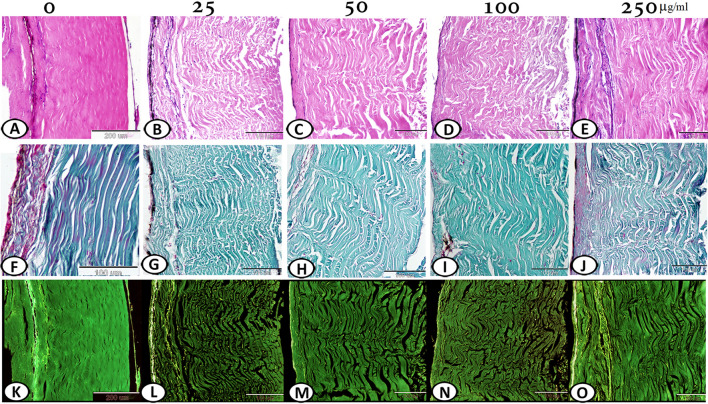
Histological analysis of Ag NPs treated Tilapia skin at concentrations of 25, 50, 100, 250 µg/mL. (**A**) Compactly arranged collagen fibers in control group. (**B**) Collagen fibers were loose showed wavey pattern at 25 µg/mL. (**C**) Collagen fibers were wavey and slightly disorganized at 50 µg/mL. (**D**) Highly disorganized collagen fibers were observed at 100 µg/mL. (**E**) Collagen fibers were compactly arranged and slightly loose at 250 µg/mL. (**F**, **K**) Normal structure of the skin components. (**G**, **L**) Moderate change in collagen intensity and organization at 25 µg/mL. (**H**, **M**) Mild change in the disposition pattern of collagen fibers and the collagen intensity at 50 µg/mL. (**I**, **N**) Marked reduction in intensity of the collagen fibers at 100 µg/mL. (**J**, **O**) Fish skin showed well-organized collagen fibers and high percentage of collagen intensity at 250 µg/mL. (**A**–**E** stained with HE, **F**–**J** stained with Crossmon’s trichrome, K–O are negative image).

The histological analysis of the untreated fish skin in the control group revealed that the dermis contained compactly arranged collagen fibers in a parallel pattern, covered with epidermis with a characteristic distribution of melanocytes (Fig. 2A). The collagen fibers were loose with a wavey pattern at 25 µg/mL (Fig. 2B), wavey and slightly disorganized at 50 µg/mL (Fig. 2C), highly disorganized at 100 µg/mL (*P* < *0.0001*) (Fig. 2D), and compactly arranged with slight looseness at 250 µg/mL (Fig. 2E).

### Histochemical analysis

**Figure 3:**
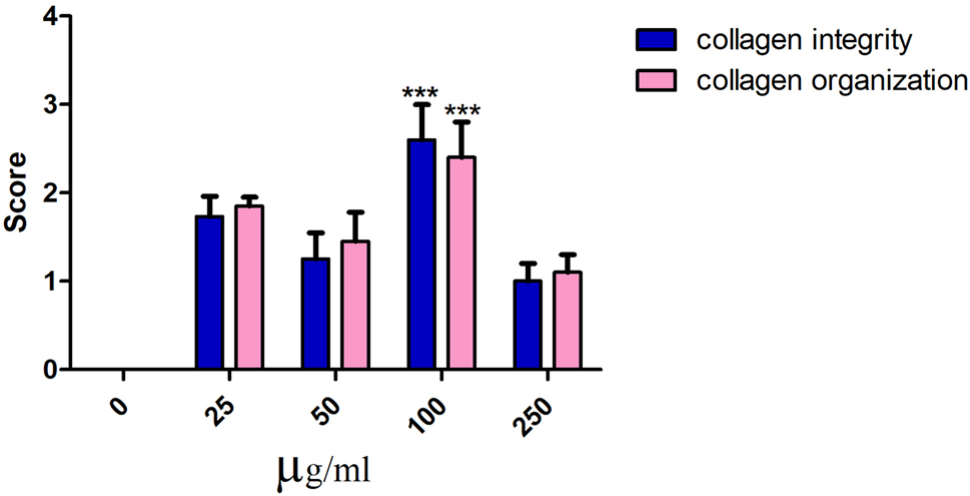
Morphometric analysis of the collagen integrity and organization in Tilapia fish skin under different concentrations of Ag NPs. The collagen integrity and organization were evaluated based on 0–3 scale as previously mentioned in the materials and methods. ***p < *0.0001*.

**Figure 4:**
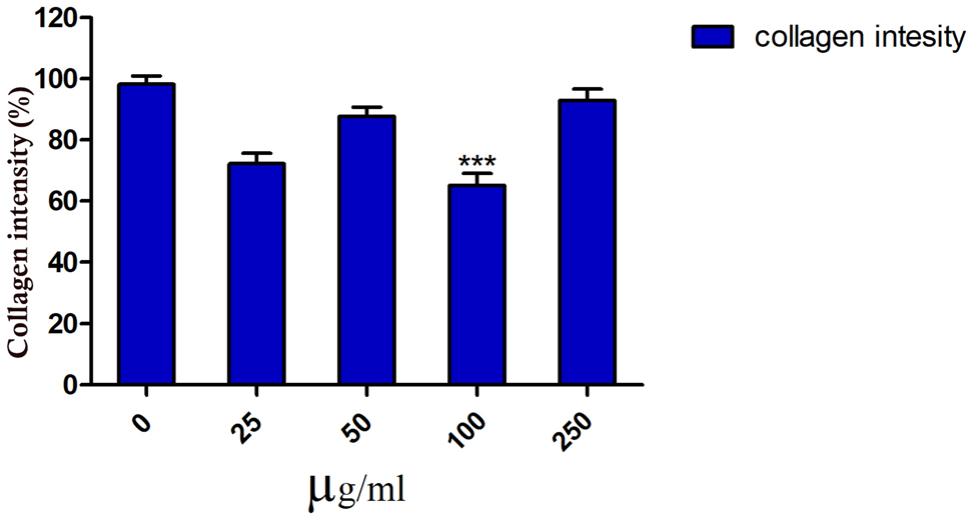
Morphometric analysis of the collagen intensity in Tilapia fish skin under different concentrations of Ag NPs. The collagen intensity was measured using ImageJ software (version 1.48v). The results are expressed as a percentage from the total number of pixels and are normalized to control-treated group. Differences were evaluated using one-way ANOVA. ***p <  *0.0001*.

This study also examined the collagen fiber organization in the Ag NPs-treated and untreated groups by staining the skin samples with Crossmon’s trichrome. Collagen intensity was measured in the fish skin using ImageJ (version 1.48v). There was a moderate change in collagen intensity and organization at 25 µg/mL (Fig. 2G and L) compared with the control group (Fig. 2F and K). A mild change in the disposition pattern of collagen fibers and collagen intensity was recorded at 50 µg/mL (Fig. 2H and M). However, there was a significant (*p* < *0.0001*) reduction in the intensity of the collagen fibers at 100 µg/mL (Figs. 2I and N and 3). The fish skin showed well-organized collagen fibers and a high percentage of collagen intensity at 250 µg/mL, compared to the control group (Figs. 2J and O and 4).

## Discussion

Tilapia skin has been used as an occlusive dressing for burn and wound healing because of its high content of collagen and homogeneity to human skin^1–3^. Tilapia skin grafts cannot be applied to the skin before being sterilized. The sterilization process of the Tilapia skin grafts should be a dual action of an efficient antimicrobial effect and a preserving effect on collagen content. Here, this study explored that immersion for 5 min in 250 µg/mL Ag NPs solution could be an optimal method for the sterilization of Tilapia skin grafts achieving efficient antimicrobial, as well as collagen preserving impacts. Moreover, this method is time-saving and economical, which makes it reliable, feasible, and reproducible in clinical applications.

Infections caused by bacteria, fungi, and viruses are serious issues in the aquaculture sector^19^. Antibiotics are the commercially accessible approach for treating infections; nevertheless, overuse over time leads to the evolution of resistant strains, rendering antibiotic treatments ineffective and failed the treatment^20,21^.

Many researchers have utilized silver nanoparticles for controlling microbial pathogens such as controlling various strains of the pathogenic bacteria *Escherichia coli*^22,23^ and, inhibiting the pathogenic bacteria; *Bacillus cereus, Staphylococcus aureus, Escherichia coli*, and *Salmonella typhimurium* effectively using silver nanoparticles^24^. Also, it was effective against pathogenic *Candida albicans, C. glabrata, C. geochares*, and *C. saitoana*. Soliman et al.^25^, recorded the high antimicrobial activity of silver nanoparticles against *Candida* sp., *Streptococcus* sp., *Bacillus* sp., *Staphylococcus* sp., *Shigella* sp., and *E. coli*. Mathivanan et al.^26^, utilized silver nanoparticles to control *P. fluorescens, Proteus mirabilis, E. coli, B. cereus and S. aureus*. Similar findings were found in this study, especially at the higher concentration (250 µg/mL) of Ag NPs exhibiting efficient antimicrobial impact.

Silver nanoparticles disrupt the respiratory chain of the targeted bacterium. During this suppression of the respiratory chain via respiratory enzymes, silver ions may interact with the thiol enzyme group, resulting in ROS^27,28^. ROS, as a significant factor impacting oxidative stress, can damage bacterial cell macromolecules such as protein synthesis and alteration, enzyme inhibition, lipid synthesis, and bacterial cell DNA and RNA oxidation and damage. ROS can induce cell death at high levels, but it can also cause serious damage or mutation to the DNA of bacterial cells at low levels^29^.

Palmer et al.^30^, indicated that all sterilization techniques can significantly affect both the physical properties and biological activity of collagen. Therefore, the selection of a safe sterilization method is a challenge.

Ethylene oxide (EtO) has been used to sterilize collagen-based wound dressings. However, EtO has some limitations; it may produce toxic residues, acts as a carcinogenic agent and is not suitable for materials stored in fluidic medium^31^. Moreover, EtO irreversibly alkylates amino acid residues, possibly affecting many properties of collagen such as the number of free amine groups and the degradation rate^32^. Huang et al.^33^, reported that skin sterilization with peracetic acid causes looseness in the pattern of collagen fibrils. Alves et al.^2^, recorded changes in collagen composition and organization in skin samples treated with chlorhexidine and sequential glycerol concentrations.

In this study, silver nanoparticles represent identical preservative of collagen integrity, especially at 250 µg/mL concentration. Concerning the collagen deposition, it was noted that it was higher at 250 µg/mL, compared to other concentrations. Cameron et al.^34^, and Gong et al.^35^, found that increasing the concentration of the Ag NPs as a percentage was associated with a higher deposition of collagen and promoted macrophage migration for discriminatory remodeling.

Many previous investigations indicated that the usage of silver as widespread bandages can reduce inflammation and scarring, potentially stop bacterial outgrowth and enhance the healthy process, inducing remodeling in the wound area^36^. This process occurs through the direct expression of certain growth factors, which leads to vasculogenesis, re-epithelialization, and deposition of collagen fibers. Moreover, silver nanoparticles can elucidate the transformation of the fibroblast to myofibroblast, which is responsible for the contraction of the wound and speed up the course of healing, and similarly can stimulate keratinocytes to proliferate in the injured area^37,38^.

## Conclusions

Ag NPs solution at the concentration of 250 µg/mL could be considered a reliable and feasible method for the sterilization of fish skin grafts by immersion for 5 min before application on human skin with an effective antimicrobial effect and a less disrupting impact on collagen content.

## Materials and methods

### Ethical approval

The National Ethical Committee of The Faculty of Veterinary Medicine, Assiut University, Assiut, Egypt, has approved all the procedures in this study under the Egyptian bylaws and OIE animal welfare standards for animal care and use in research and education.

### Synthesis of silver nanoparticles (Ag NPs)

In a typical synthesis process, a solution (A): dissolve 1 wt% of starch (Purity 98%, algomhoria, Egypt) in distilled water with the assistance of heat until obtaining a clear solution. A solution (B): dissolve 8 wt% of silver nitrate (Purity 99%, Sigma-Aldrich, USA) in distilled water in a dark container. The solution (A) was then mixed with the solution (B) in a ratio of 10:1. The mixture was transferred to a sterilized autoclave at 80 °C for 5 min. After cooling down to room temperature, the resultant solution was stored in the refrigerator for further usage.

Different silver nanoparticles concentrations specifically 25, 50, 100, and 250 µg/mL were prepared, where the concentration was adjusted using the equation:

$${C}_{1}\times {V}_{1}={C}_{2}\times {V}_{2}$$

where C_1_, C_2_, V_2_, and V_2_ refer to the initial and final concentrations and volumes, respectively.

### Tilapia skin collection

Tilapia skins were collected from fresh Nile tilapia (*Oreochromis niloticus*) (weigh: 520 ± 40 gm; standard length: 17 ± 3 cm), obtained from tanks of The Aquatic Medicine Unit, Faculty of Veterinary Medicine, Assiut University, Assiut, Egypt*.* Fish were euthanized physically by decapitation. Fish skins were dissected from the underlying tissues after the removal of fish scales and divided into 5 × 3- cm strips in sterile normal saline.

### Sterilization procedure

Strips of fish skin (n = 5) were randomly allocated to be immersed in silver nanoparticles (Ag NPs) solution at different concentrations of 25, 50, 100, and 250 µg/mL for 5 minutes (min) each. The Ag NPs-treated skin strips underwent bacteriological and histological evaluation following each treatment.

### Microbiological evaluation of Ag NPs-treated fish skin

The dilution plate method was used for counting the microbial cells attached to the fish skin following Downes et al.^39^, Using a sterilized swab needle, fish skin (3 × 3 cm^2^) was swabbed before (control) and after each treatment and suspended in 10 mL sterilized saline solution and mixed using a vortex (VELP Scientifica, UK) for 2 min. Different types of media were used for microbial counting and identification. Nutrient agar and MacConkey agar media were used for counting the total aerobic bacteria^40^, yeast malt extract medium used for yeast counting^41^, and potato dextrose agar medium for filamentous fungi^42^. One milliliter of the solution was transferred into sterilized Petri dishes (three replicates for each) and a sterilized isolation medium of 45 °C was poured into the dish. The plates were left for solidification in sterile laminar flow. Plates were incubated for 24 h at 35 ± 1 °C for bacteria, 3 days at 28 ± 1 °C for yeasts, and 7 days at 28 ± 1 °C for filamentous fungi. The number of developed colonies was calculated as CFU/Cm^2^. Developed colonies with morphological character differences, such as colony color, size, pigmentation, and edge, were sub-cultured and purified using the same media. Bacterial isolates were identified using the biochemical tests as described by Bergey’s Manual of Systematic Bacteriology^43^. Yeast isolates were identified according to the phenotypic characteristics including shape, color, surface, margin, budding, the formation of pseudohyphae, mycelium, arthroconidia, ballistoconidia, chlamydospore, endospore, germ tube, glucose fermentation, and carbon source utilization^41^.

### Histological evaluation of Ag NPs-treated fish skin

Specimens (0.5 × 0.5 cm) were collected from the Ag NPs-treated and untreated (control) fish skins and fixed in Bouin’s solution for 22 h. After proper fixation, specimens were processed through dehydration in ethanol, clearance in methyl benzoate, and embedded in paraffin wax. Paraffin sections of 5 µm were obtained and were stained with Harris hematoxylin and Eosin (HX) for general histological evaluation and with Crossmon’s trichrome for evaluation of collagen content.

### Negative image study

The negative image study was conducted using CMEIAS color segmentation. Improved computing technology was used to process color images by segmenting foreground object pixels from the background with this technique is especially useful because of the complex color micrographs that existed in this study.

### Morphometrical analysis

The histological evaluation was conducted blindly on coded samples, and a comparison was performed with the sections from the untreated- and treated groups. Histological evaluation to assess the integrity and organization of collagen fibers was based on 0–3 scale^44,45^. Collagen integrity scores: 0 = continue, long fiber, 1 = slightly fragmented, 2 = moderately fragmented, 3 = severely fragmented. Collagen organization scores: 0 = compact and parallel, 1 = slightly loose and wave, 2 = moderately loose, wavy, and cross to each other, 3 = no identifiable pattern. Using threshold area fraction determination, the percentage of the collagen-positive area was calculated using ImageJ (ImageJ Software Version 1.48v)^2^. The amount of collagen was reported as a percentage of the total number of pixels in the optical view as a percentage and expressed as mean ± SEM.

### Statistical analysis

Statistical analyses were performed by one-way ANOVA using GraphPad Prism software version 5.03 (GraphPad Software Inc., La Jolla, CA, USA). p-values of < 0.05 were considered statistically significant. Figures were generated using Adobe Photoshop CS6 and Prism 5 version 5.03.

### Ethical approval

All the procedures in this study have been approved by The National Ethical Committee of The Faculty of Veterinary Medicine, Assiut University, Assiut, Egypt, under the Egyptian bylaws and OIE animal welfare standards for animal care and use in research and education. We did not use any human skin. This study was conducted in compliance with the ARRIVE guidelines. All methods were performed in accordance with relevant guideline and regulations.

## Data Availability

All data generated or analyzed during this study are included in this published article.
